# Strengthening of banana breeding through data digitalization

**DOI:** 10.1093/database/baz145

**Published:** 2020-04-11

**Authors:** B Vignesh Kumar, S Backiyarani, A Chandrasekar, S Saranya, D Ramajayam, M S Saraswathi, P Durai, S Kalpana, S Uma

**Affiliations:** Crop Improvement Division, Indian Council of Agricultural Research - National Research Centre for Banana, Thogamalai Road, Thayanur Post, Tiruchirapalli - 620 102, Tamil Nadu, India

**Keywords:** Banana breeding tracker, QR-based BBT, Real-time monitor, improvement

## Abstract

Improvement of edible bananas (a triploid and sterile crop) through conventional breeding is a challenging task owing to its recalcitrant nature for seed set, prolonged crop duration. In addition, the need of huge man power at different stages of progeny development and evaluation often leads to mislabeling, poor data management and loss of vital data. All this can be overcome by the application of advanced information technology source. This ensured secure and efficient data management such as storage, retrieval and data analysis and further could assist in tracking the breeding status in real time. Thus, a user-friendly web-based banana breeding tracker (BBT) has been developed using MySQL database with Hypertext Preprocessor (PHP). This BBT works on all operating systems with access to multiple users from anywhere at any time. Quick responsive (QR) code labels can be generated by the tracker, which can be decoded using QR scanner. Also for each and every updated progress in breeding stages, a new QR code can be generated, which in turn reduce labeling errors. Moreover, the tracker has additional tools to search, sort and filter the data from the data sets for efficient retrieval and analysis. This tracker is being upgraded with phenotypic and genotypic data that will be made available in the public domain for hastening the banana improvement program.

## Introduction

Banana (*Musa* sp.) is one of the economically important fruit crops of the tropics and subtropics (1) regions that serves as a staple food in Africa and Pacific countries (2). The contemporary banana varieties have evolved naturally through intraspecific and interspecific crosses among *Musa acuminate* and *Musa balbisiana*. However, most of the commercial cultivars are highly susceptible (3, 4) to the new and emerging pest and diseases (5), which resulted in serious fluctuation in banana production. Several literature evidence stated that certain wild or related species or some landraces are resistant/tolerant to abiotic and biotic stresses (6), which cannot be commercialized owing to their seediness and poor yield. Conversely, these resistant sources can be exploited for improving the commercial cultivars through conventional breeding complemented with marker-assisted selection.

Success of conventional breeding method for the improvement of banana is very limited owing to its recalcitrant nature for the seed set and germination (7). These can be overcome by developing intermediate breeding lines such as tetraploids (4×) or improved diploids (2×) (8, 9) and by employing embryo culture or embryo rescue techniques (10, 11). Banana breeding program is a time-consuming process that involves huge manpower to complete the breeding procedures, which makes it cumbersome in tracking the status of hybridization details. Maintaining the pedigree information on intermediate lines will facilitate the breeders for further selection of parents and also in understanding the genetic architecture of plants through present-day next-generation sequencing platforms (12, 13). Moreover, genomic selection (GS), a new approach for improving quantitative traits in large plant breeding populations requires marker data along with phenotypic and pedigree information (14). This emphasizes the importance of maintaining the accurate breeding information for the present and future scientific applications in any crop improvement programs.

The conventional method of storing the breeding information in field books often leads to manual errors during data entry and risk of missing datasheet/book etc. An alternate approach to minimize the errors, for easy maintenance and retrieval, data sharing among breeders and other end users is essential. Further to avoid errors that occur during transcribing or updating the labels at each stage of the breeding program, information needs to convert in the form of quick responsive (QR) codes. Hence, there is an urge for an information system to develop the database that can be used to feed, retrieve, analyze and share data like MusaBase (https://musabase.org), MGIS (https://www.crop-diversity.org/mgis/), ProMusa (http://www.promusa.org/) and MusaNet (http://www.musanet.org/) (15). Breeding tracker has been developed for potato (15), cotton (16), apple (17), banana (https://musabase.org) and are being used in their respective breeding programs. But none of these trackers have the facility to develop digitalized labels. Therefore, an attempt was made to develop a user-friendly digital-based database to track the banana breeding program with QR code application, which will work on all operating systems (OSs).

## Materials and Methods

### Database construction

A backend datasheet was created using MySQL database with eight tables, which represents the steps involved in the breeding program and altogether have 53 pre-defined fields to feed the banana breeding information through Hypertext Preprocessor (PHP) forms. All the developed PHP forms were linked with MySQL database through codes using Microsoft visual studio codes (source code editor) i.e. this tracker is constructed with PHP-MySQL-PHP scheme.

### Database implementation

**Figure 1:**
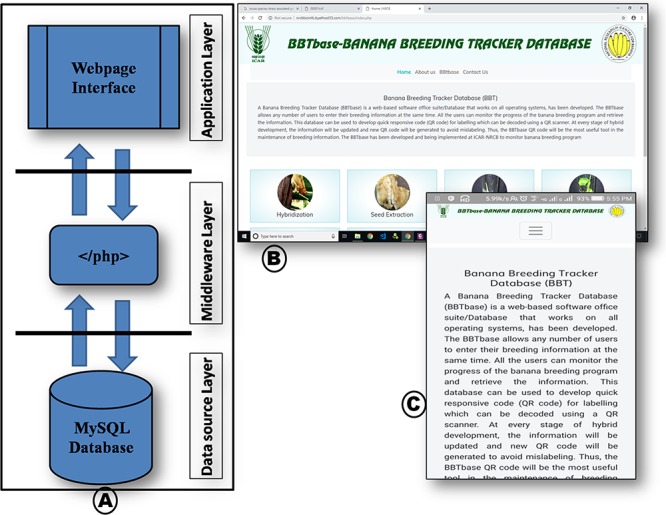
(A) Database architecture and schema, (B) RWD database in computer chrome browser and (C) smartphone chrome browser.

This tracker can run on any OS such as Windows, Mac, Chrome, Android, iOS and Linux. To facilitate the user, the tracker was developed with responsive web design (RWD) approach, which enables efficient performance of the web page on a variety of devices. The tracker architecture is shown in Figure 1.

**Figure 2:**
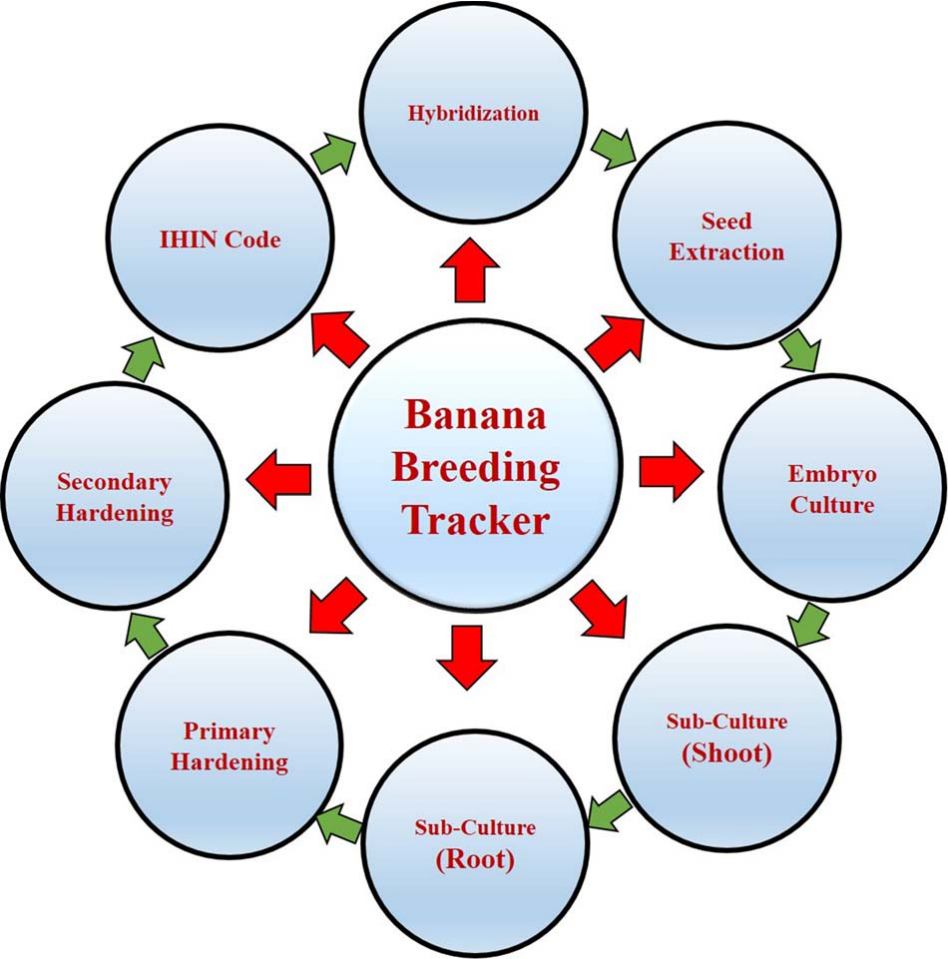
Schematic architecture of BBTbase and breeding stages.

**Figure 3:**
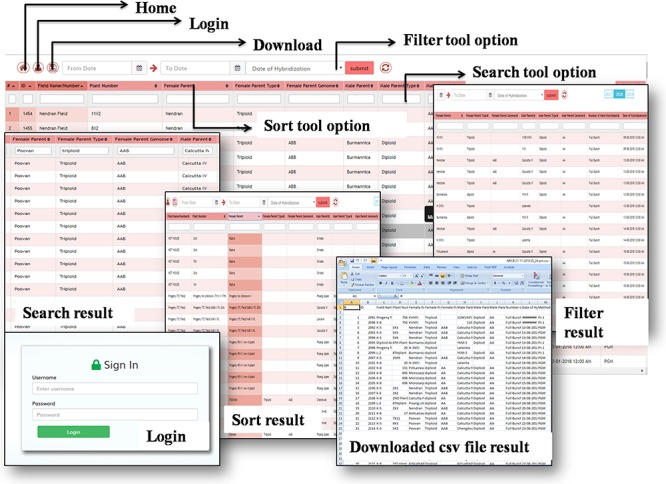
Search, sort, filter and login and download tool options in the BBT and their outcome. The user can access their data by using (A) filter, (B) sort, (C) search and (D) browsing options. Pop-ups showing some examples of (J) filtered, (H) searched, (L) sorted data and (K) downloaded data as csv file. The obtained result can be downloaded by (G) save file option.

### Database interface

#### Interoperability

Banana breeding tracker (BBT) was developed with the integration of MySQL, PHP and QR code. This can be accessed online through the link http://nrcbbioinfo.byethost33.com/bbtbase/index.php, and the data can be entered using PHP data collector form in the pre-defined fields. The fed details will be directly saved into the secured backend, and the same will be updated whenever the new data are fed.

To label any intermediate breeding materials developed at different stages of the breeding program, the necessary information available in the tracker will be converted into QR codes using a QR code generator. Thus, in BBT, the hybridization details are mapped as QR code 1. The data on seed extraction is mapped as QR code 2 along with information of QR code 1. QR code 3 contains data on embryo culture together with the information of QR code 2. Shoot and root induction data on each embryo culture are mapped as QR code 4 and 5, respectively, along with selected information of QR3. The QR codes 6 and 7 contain additional data on primary and secondary hardening of hybrid progenies, respectively. QR code 8 contains National Research Centre for Banana Individual Hybrid Identification Number (NRCB IHIN) code with 10 digits alpha-numerical letters. For example, ProKVPJ17/002—here `Pro’ denotes Progeny, `KVPJ’ first two letters denote the female parent and the next two letters denote the male parent i.e. it represents the parental combination [Female—Karpooravalli (KV) and male—Pisang Jajee (PJ)], `17’ refers to the year of hybridization and last three digits (002) for progeny number produced with same parental combination in the same year. For retrieving the information as per the user’s privilege, the search, sort and filter options have been provided in all the fields of the tracker along with date wise filtering option.

## Result and Discussion

Retrieval of breeding information using traditional method of data maintenance such as field notebooks is cumbersome and often leading to human errors like mislabels that deteriorate and disorient the quality of the breeding information (6, 18). This has been overcome by digitizing the entire information that can be converted into labels with QR code for easy retrieval and tracking.

The information stored in the database at each stage of the breeding program can be viewed and retrieved in the form of QR codes and can be printed using handheld devices. The printed QR codes can be used as labels for tagging the breeding material both in field and laboratory. Any user can access the information on QR code by scanning the QR codes using the hand-held data capture devices such as barcode or QR code scanner or by using smartphones that has code scanning feature.

Details of pollinated hands that can be retrieved from QR code 1 will facilitate the seed extractor to differentiate the pollinated hands from un-pollinated hands, and thereby pollinated hands alone will be subjected to seed extraction. This saves unnecessary consumption of manpower, and the fruits of un-pollinated hands can be used for any other analyses or consumption. Adam *et al.* (19) reported that prolonged period of seed storage reduces the germination percentage, which emphasized that the extracted seeds should be subjected to germination within a short period of time in order to increase the germination percentage. The information about the date of bunch harvest and seed extraction provided in the QR code 2 shall facilitate the prioritization of the crossed seeds for embryo culture.

Viable embryos germinate within 9–10 days could extend up to 30 days, beyond which the embryo regeneration is limited (11, 20). Thus, QR code 3 has been provided with additional information on the date of embryo initiation that makes the breeder to decide whether to maintain the culture or not. In general, embryos produce healthy shoot and ramified roots within 60 days of initiation and occasionally need additional sub-culturing in shoot and root induction medium. QR codes 4 and 5 have been created to record such information about the date of sub-culturing, shoot and root induction, respectively. QR code 6 provides details regarding the date of shifting of rooted plantlets to primary hardening where it is normally maintained for 3–4 weeks. QR code 7 contains the date of shifting of primary hardened plants to secondary hardening with NRCB IHIN number. QR code 8 contains only the NRCB IHIN number, which will be used until the end of the evaluation of hybrids for their agronomic and others traits of interest in the field trials.

The germination percentage differs based on the fruit maturity at the time of harvest, physiological age of the seed, storage conditions, media composition, parental combination etc. (10, 21, 22). Thus, understanding the reason for successful banana breeding program is of paramount importance that helps in selecting the suitable protocol to enhance the success rate. In this progress, BBT will be an important tool to identify and formulate the best strategies in the breeding program. This can be achieved by retrieving the banana breeding information stored in the MySQL database at each stage and/or at the final stage. Since the tracker contains enormous breeding data, it has been provided with search, sort and filter options for easy handling and retrieval.

The users can use the search options in various combinations of fields to retrieve desired information. A sort tool option is also provided in all the fields for any end user to sort the data. Further, in order to collect the information of each breeding stage on date wise, this tracker is added with the filtering option in the fields wherever the dates are fed. Using these search, sort and filter options, one can access the stage-wise output of the hybridization program. Thus, the data can be retrieved as per the user requirement, which will facilitate the researcher to identify the best parental combinations, favorable seasons, microclimate for seed set and mode of pollination, and additionally it provides an option to select the optimal media composition for embryo culture etc.

Moreover, to browse the breeding information on year wise, a user-friendly button option has been provided at the right side corner of the tracker to extract the required information from the huge breeding data and facilitate the principal breeder to track the breeding status in real time. Besides, in the result page, the user can effortlessly calculate and compare the data regarding the number of embryo initiated, embryos regenerated into plantlets, number of hybrids going for field evaluation etc. using automatic calculation method, while searching and sorting the information. The login functionality has also been provided to download the data in the form of CSV file, which can be easily shared among the partners and co-workers.

### Utility

The tracker facilitates the banana breeder for onsite digital data recording. Since the breeding data are in the digital form, it offers the following: (i) easy retrieval of data and statistical analysis, (ii) user can track and monitor the breeding program in real time and (iii) cloud storage—for safe data storage mechanism.

Moreover, it also provides the necessary information in the form of QR and thereby minimizes the labeling error. The data entered in the BBT can be viewed in a simple way to assess the rate of success at every stage of the breeding program. It will facilitate the analysis of voluminous data to understand the reasons for poor seed set and embryo germination percentage and thereby allows the planning of strategies that are armed for the production of more banana hybrids. It provides eco- (paper-free) and user-friendly environment that facilitates long-term record maintenance.

### Validation of BBT

The BBT was validated with the inputs from 2000 crosses using diverse banana parents at each breeding stage. All the fed data could be successfully retrieved using search, sort and filter options. The QR code generated labels were also verified using scanners and being put to continuous use by the partners involved in the banana breeding program.

## Conclusions

This BBT is available online and compatible with CSS3 enabled browsers like Google Chrome, Mozilla Firefox, Microsoft Edge, Internet Explorer, Safari, Opera etc. Altogether, this tracker will be intuitive to accelerate any breeding program.

### Future perspectives

The future versions are being created with the addition of new datasheets for feeding phenotypic and genotypic information of germplasm and progenies to hasten the development of new varieties and trait-specific markers.

### Declarations

#### Availability

BBT can be easily accessible through the following URL: http://nrcbbioinfo.byethost33.com/bbtbase/index.php. All source code is available on the BBT GitHub repository (https://github.com/directornrcb/BBT-Banana-Breeding-Tracker) and accepted by FAIRsharing (https://doi.org/10.25504/FAIRsharing.EtYkWo).
